# Vulnerability and Pediatric Pain

**DOI:** 10.1002/pne2.70020

**Published:** 2026-04-20

**Authors:** S. van Rysewyk, D. Harrison, A. Harvey, E. Ilhan

**Affiliations:** ^1^ Department of Philosophy and Gender Studies School of Humanities, University of Tasmania Hobart Australia; ^2^ Department of Nursing School of Health Sciences, Faculty of Medicine, Dentistry and Health Sciences, The University of Melbourne Melbourne Australia; ^3^ School of Nursing, Faculty of Health Sciences, University of Ottawa Ottawa Canada; ^4^ Neurodisability and Rehabilitation, Murdoch Childrens Research Institute Melbourne Australia; ^5^ School of Health Sciences and Nursing Faculty of Medicine, Health and Human Sciences, Macquarie University Sydney Australia; ^6^ Grace Centre for Newborn Intensive Care, The Children's Hospital at Westmead Sydney Australia

**Keywords:** cerebral palsy, communication needs, intellectual disability, neonate, pain, preterm baby, vulnerability

## Abstract

Healthcare disparities amplify vulnerabilities in infants and children. In the context of pain, vulnerable individuals are those whose pain often goes under‐appreciated, ‐recognized, or ‐treated. Vulnerability to pain arises due to multiple and interacting sources including inherent vulnerability, situational vulnerability, and pathogenic vulnerability. Some groups are more vulnerable to pain than others, which will be the focus of this article: sick and preterm newborns, and children with intellectual disabilities and complex communication needs, including children with cerebral palsy. In this conceptual review, we highlight vulnerabilities to pain in these vulnerable populations and identify how we have obligations to minimize harm experienced by sick and preterm newborns, and children with developmental disabilities. Although pain is a universal experience, not everyone has equal and fair access to the necessary services for adequate pain management. This is particularly the case for sick and preterm babies who are exposed to large numbers of painful procedures during their hospitalization, and individuals with intellectual disability and complex communication needs, who have limited language and/or speech capacity to self‐report pain. Therefore, explicit reflection on vulnerability reinforces a collective responsibility to provide effective assessment and management of pain in particularly vulnerable pediatric populations.

## 1 Introduction

Concern for human vulnerability is at the heart of pain management, driving our efforts to understand, identify, and alleviate pain. In 2019, the International Association for the Study of Pain (IASP) convened a task force to improve pain management in people unable to articulate their pain in a way that health professionals can understand and/or whose pain is underestimated [1]. The taskforce identified older persons, infants and young children, individuals with cognitive impairments or psychiatric disorders, and pain in survivors of torture, as “belonging to the most vulnerable”. The aim of this paper is to advance a theoretically grounded framework for understanding vulnerability to pain in pediatric contexts. Drawing on existing ethical, philosophical, and empirical scholarship, we apply this framework to two groups at particular risk of harm: sick and preterm newborns, and children with developmental disabilities and complex communication needs. The paper examines three intersecting sources of vulnerability: inherent, situational, pathogenic, and considers how each shapes the experience and management of pain in these populations. Specifically, we ask: (1) how the concept of vulnerability can explain why sick and preterm newborns, and children with developmental disabilities and complex communication experience unrecognized or undertreated pain; (2) how clinical, social, and institutional practices may contribute to, or alleviate, these vulnerabilities; and (3) how a relational understanding of vulnerability can inform ethical and professional obligations to minimize harm. By addressing these questions, the paper seeks to translate theoretical insights about vulnerability into practical considerations for pain research, clinical care, and health policy.

### 1.1 Defining Vulnerability

Vulnerability is widely understood as a universal condition that binds together all human beings (e.g., see references [2, 3, 4, 5]). Vulnerability derives from the Latin *vulnus*, meaning “wound”, and to our capacity to feel pain, develop disease or sickness, which are inherent to human embodiment [5]. “Embodiment” refers to the fact that each human “has” a physical body with a propensity to feel pain develop disease and sickness, succumb to impairment, disability, and death. It should be noted that in extremely rare cases, some individuals have a genetic disorder which causes pain insensitivity and pain indifference called congenital insensitivity to pain. Individuals with congenital insensitivity to pain may not “feel” pain but are still vulnerable to harm due to their increased risk of injury and repeated injuries which may result in delayed healing.

The sociality of human life is an important dimension of vulnerability. As embodied, social beings, we depend on the care and support of other people, and we are vulnerable to the actions of others. As vulnerability is a condition of our humanity, we are all equally vulnerable. But some people are more vulnerable than others. How do we determine levels of vulnerability to pain? Why does vulnerability generate obligations? And why do we have obligations to the most vulnerable? [6].

### 1.2 Principle of Protecting the Vulnerable

Goodin [7] views vulnerability in terms of harm and one's relation to others. First, to be vulnerable is that one's needs or interests are susceptible to harm. Second, we are vulnerable to other people. Other people can threaten my needs or interests. This is the principle of protecting the vulnerable. Vulnerability generates obligations because we have the capacity to act to prevent harm, or to protect the needs and interests of people who are vulnerable to our actions and choices [7]. Indeed, the ethical conduct of human research, such as pain research, is based on this principle—we are obligated to minimize harm imposed on individuals [7] as a result of the design, execution, and dissemination of research [8].

What generates an obligation is not some special intellectual insight that only I have, but your dependency on me, which makes you vulnerable to my actions and choices. Therefore, this obligation is based on social relationships. The relational nature of vulnerability has implications for our obligations in society. The more vulnerable you are to me in terms of which I can harm your needs or interests, the greater is my inherent responsibility to protect you. For example, an infant or child's needs or interests are especially vulnerable to harm by the actions of adults and by extension, the systems in which society functions (e.g., healthcare).

### 1.3 Vulnerability, Harm, Exploitation

Goodin's [7] principle highlights an important connection between vulnerability, harm, and exploitation. The fact that not everyone is equally vulnerable creates opportunities for more powerful persons to take unfair advantage of or exploit more vulnerable persons, especially where the powerful party has discretionary control over resources that the other needs and cannot obtain elsewhere. Goodin's principle obligates more powerful parties in unequal relationships to be vigilant against misuse of their power, authority, or privilege to take unfair advantage of others, and to protect those who are vulnerable to them. For example, vulnerable individuals may be at risk of harm by exploitation in the research context—placebo‐controlled trials of sucrose in the management of single painful procedures (e.g., heel lance) in sick infants hospitalized in NICUs continue to be conducted, despite the lack of clinical equipoise [9, 10, 11, 12, 13]. Such instances are not uncommon, particularly in research on vulnerable populations such as infants who are unable to communicate, or act as autonomous decision‐makers. In this case, enrolment in clinical trials that lack clinical equipoise prevents access to essential care (i.e., pain relief) in a vulnerable group (i.e., sick and preterm babies). Hence, research participants may not only *be* vulnerable, but they *become more* vulnerable because of unjustifiable (i.e., due to the lack of clinical equipoise) research processes. Some groups, such as children with developmental disabilities, may have “distinct vulnerabilities as research participants…[and are] more‐than‐usually vulnerable to various forms of discomfort and stress” (p. 75) [14].

### 1.4 Vulnerability and Autonomy

There is also a connection between vulnerability and autonomy. Autonomy is the idea that people are independent, self‐sufficient, and able to meet personal needs or interests. Respect for patient autonomy is a cornerstone of medical ethics and an important principle for health professionals [15]. Unrelieved pain may so consume the patient that it impinges on a patient's ability to make choices regarding care and obligates health professionals to treat such pain to restore autonomy. Indeed, vulnerability is associated with a dependency on others and is heightened in situations when a person cannot communicate their pain. However, because it is difficult to apply the concept of autonomy to sick and preterm babies and children with complex developmental disabilities, vulnerability may be better articulated as the “identifiably increased likelihood of incurring additional or greater wrong” (p. 195) [16], regardless of whether consent is valid or whether an individual is able to act autonomously. In other words, the capacity to act autonomously is not a condition for protecting the vulnerable. Luna [17] expands on this by stating that vulnerability is not a category, but rather a dynamic and relational “…situation under analysis” (p. 89) and always context dependent. Therefore, an individual's vulnerability to pain may change from moment to moment, particularly when communication is hindered as is the case for sick and preterm infants and children with developmental disability and complex communication needs.

### 1.5 Three Interacting Sources of Vulnerability to Pain

Hospitalized preterm and sick infants and children with developmental disabilities are especially vulnerable to pain. For sick infants, increased vulnerability to pain relates not only to the immaturity and increased sensitivity to pain but to the following four factors:

- The large number of repeated painful and stressful procedures required for medical care;
- Inconsistent use of effective recommended pain management strategies;
- Separation from and exclusion of parents from NICUs during painful procedures;
- Adverse effects of pain: association between the number of painful procedures and poorer neurocognitive outcomes.

For children with developmental disabilities, vulnerability to pain is exacerbated in the presence of complex communication needs, a broad term that describes people with severe speech, language, and communication impairments causing them difficulty communicating in everyday life, including self‐reporting pain. Children with disabilities may have pain go unnoticed if their pain response is atypical or unrecognized [18]. In addition, children with disabilities may express pain in ways other than speech, including the use of gestures or behavioral changes. The increased vulnerability to pain experienced by preterm and sick infants and children with developmental disabilities can be understood as arising from three interacting sources: inherent vulnerability, situational vulnerability, and pathogenic vulnerability (Table 1). These sources of vulnerability are individually explored in the following sections.

**TABLE 1:** Interacting sources of vulnerability and clinical examples in pediatric pain.

Vulnerability type	Definition	Clinical exemplars in pediatric pain
Inherent	Vulnerability arising from intrinsic characteristics (biological, developmental, or dependency‐related) that make the individual unable to avoid harm without protection.	Hospitalized neonates undergoing frequent painful procedures without evidence‐based pain management. Children with developmental disabilities who cannot verbally communicate pain.
Situational	Context‐dependent vulnerability created by circumstances of illness, treatment, or environment that limit autonomy, or amplify distress.	A previously healthy child immobilized post‐surgery and dependent on caregivers for pain relief. Hospitalized newborns and children separated from parents during painful interventions.
Pathogenic	Vulnerability produced or intensified by social, structural, or institutional practices, including discrimination, exclusion, or policy failures.	Parental exclusion from NICU during procedures, despite evidence of parent‐involved strategies including analgesic benefit of skin‐to‐skin care. Children with communication disabilities denied validated pain self‐report tools due to systemic underinvestment in resources.

### 1.6 Vulnerability to Pain Arising From Our Human Condition—Inherent Vulnerability to Pain

Inherent vulnerability to pain refers to sources of vulnerability that are inherent to the human condition. They arise from our embodiment, the human need for support and protection, and the impact of trauma or persistent noxious experiences on our quality of life. A person's inherent vulnerability to pain varies depending on a range of factors, such as age, sex assigned at birth, gender identity, health status, or level of disability. Overall, women are more likely to report chronic pain than men [19]. Chronic pain is generally more common with increasing age [20]. Inherent vulnerability to pain also varies based on a person's resilience, ability to cope, and the social support available (e.g., [21]). While some groups may be inherently vulnerable [22], there is within‐group variation in the severity of susceptibility to pain. For example, while all infants are susceptible to harm, preterm and sick infants are more susceptible to pain by virtue of their situational vulnerability [22, 23].

### 1.7 Vulnerability to Pain Caused by a Particular Situation—Situational Vulnerability to Pain

This vulnerability to pain is context‐specific. It is caused or exacerbated by the particular situation of a person or social group. It can be short term, intermittent, or enduring.

#### 1.7.1 Sick and Preterm Infants

Vulnerability to pain is exacerbated situationally by critical illness. Sick and preterm infants require large numbers of repeated painful and stressful procedures throughout their hospitalization for treatment and monitoring, with heel lances being the most frequently reported invasive painful procedure [24]. The earliest published newborn pain prevalence study [25] reported that on a cohort of 54 sick newborns who underwent 3283 skin‐breaking painful procedures, an average of 61 painful procedures per infant, with over half of these (*n* = 1849) being heel lances. The true number of procedures was likely higher as unsuccessful attempts were not counted, and procedures such as nasogastric tube insertion were not reported. Such reports have continued to be published since 1995. A systematic review of 18 observational studies of painful procedures in NICUs, published between 1995 and 2014, included data for over 3000 infants [26]. The total number of painful procedures recorded ranged from 6832 up to 42 413, a mean of 7.5–17.3 painful procedures per neonate per day, with heel lance procedures the most frequently recorded. Most procedures were performed with no pain treatment. More recently, Bueno and colleagues included 35 studies which included 5587 neonates, reporting on frequency and nature of painful procedures in NICUs published over the last 27 years [24]. Similar to the earlier review, the majority of procedures were performed with no pain treatment. In addition, these large numbers of reported procedures probably underestimated the true number of painful procedures as not all procedures are documented and repeat attempts are rarely captured. These reviews highlight the large burden of pain that sick newborns experience during a critical period of neurodevelopment.

Preterm and sick neonates are especially vulnerable to adverse long‐term developmental outcomes as a result of pain [27]. A crucial aspect of vulnerability for preterm and sick neonates is the growing evidence of the association between the cumulative number of tissue breaking painful procedures with adverse early and later developmental outcomes affecting brain development and developmental and cognitive outcomes [27, 28, 29]. The need for repeated painful procedures as part of nursing and medical care, coupled with being cared for in often very noisy environments [30], without the presence of parents to help comfort and advocate for best pain management, puts preterm and sick newborns in a state of extreme vulnerability. Strategies are therefore urgently needed to improve pain treatment during painful procedures for premature and sick newborns.

#### 1.7.2 Children With Developmental Disabilities

Pain is highly prevalent for children and young people with developmental disabilities. In cerebral palsy, the most common physical disability of childhood, up to 76% of children experience any pain type [31], and approximately one‐in‐three experience chronic pain [32]. Pain has been poorly understood, inadequately assessed, and consequently ineffectively managed in this vulnerable population [33]. Several factors may contribute to a high prevalence of pain in children and young people with physical disabilities. Management interventions including therapies, orthoses, surgeries, and procedures are common throughout childhood [32, 34, 35, 36]. The use of equipment, for example standing frames, is commonly associated with pain [37] as are musculoskeletal issues, hypertonia, and gastrointestinal issues [31, 38, 39].

Difficulty communicating or being understood can exacerbate situational vulnerability. In Australia, approximately 63% of children with cerebral palsy at 5 years of age have a speech impairment, with 41% being classed as non‐verbal [40], making them very vulnerable to harm due to not having their opinions and preferences heard [34, 36]. Children with significant communication difficulties who experience pain require alternative means, such as augmentative and alternative communication strategies (AAC) and systems, to communicate their pain in order to receive appropriate treatment [41]. In the absence of such alternative means, children with developmental disabilities cannot convey their pain and hence receive appropriate care. Children and families also articulate how important it is to understand a child's own pain descriptions, and to provide adjustments to ensure children with cognitive and communication impairments can report their pain. Many children with complex communication needs can have undiagnosed, undertreated and dismissed pain experiences, largely because they do not have the opportunity to self‐report their pain to others [34]. Pain results in reduced social and school engagement, quality of life and psychological wellbeing for young people with cerebral palsy [42]. Qualitative studies involving children and young people and their families illustrate the burden and impact pain has on the child and family, and how it is a determining feature of their lives [34, 35, 43, 44, 45]. It is perhaps unsurprising that these children are at a much higher risk of physical and psychological suffering and distress due to their under‐recognized pain [46]. Often the child is deemed to be unable to report their pain due to communication barriers and not provided the opportunity to do so [47]. In these situations, proxy‐reporting is utilized; however, this can cause biases.

### 1.8 Vulnerability to Pain Caused by Dysfunctional Relationships—Pathogenic Vulnerability to Pain

Some responses to pain can exacerbate existing vulnerabilities to pain or generate new vulnerabilities through dysfunctional relationships based on disrespect, prejudice, or abuse, or by socio‐political situations characterized by oppression, injustice, persecution, or political violence. Many groups most likely to be targeted by “pathogenic vulnerability” are subject to oppression. In the United States, for example, older adults, women, veterans, and African‐Americans are a few of the social groups who endure worse treatment for their pain than members of dominant groups (e.g., [48]).

Pathogenic vulnerability can be demonstrated by paternalistic attitudes towards vulnerable populations. For example, excluding certain groups from participating in research could increase that group's susceptibility to indirect harms due to preventing scientific advances in our understanding of pain and pain management in vulnerable individuals [49, 50]. Indeed, children with severe intellectual disabilities are more at risk of having pain, but are rarely involved in research, causing an inequity in health care and the direct and indirect benefits of being involved in research [18]. Pathogenic vulnerability may also occur in the NICU. For instance, separation from and exclusion of parents from NICUs during painful procedures puts sick newborns particularly at risk [51, 52, 53, 54, 55]. High‐quality synthesized evidence demonstrates analgesic effects of breastfeeding during procedures when possible, feasible, and culturally acceptable [56], skin‐to‐skin care (SSC), especially for preterm newborns [57], and sucrose or other sweet‐tasting solutions for preterm and term newborns [12, 58, 59]. These strategies are discussed and demonstrated in various parent‐targeted resources [60, 61, 62, 63, 64]. However, studies consistently show infrequent use of these strategies, especially parent‐led strategies [52, 65, 66], despite efforts made to embrace family‐centred care for hospitalized newborns and children [55, 67]. Harrison and colleagues reported that most mothers of sick infants who had been hospitalized in NICUs did not know about the evidence for SSC, had not used SSC during painful procedures, and were distressed about their lack of opportunity to be involved to comfort their newborns during painful procedures [52, 68, 69]. Other factors that prevent parental involvement in pain management include cultural norms, clinicians' perspectives of parents' unwillingness to be involved, a lack of facilities to accommodate parents in many NICU settings, and organizational and societal factors [55, 69]. Such factors demonstrate several potential pathogenic vulnerabilities to pain. It is clear that major shifts in coordination of NICU care at many levels are needed to enable the involvement of parents during non‐urgent scheduled painful procedures in order to minimize pathogenic vulnerabilities. Aligned with this conception of pathogenic vulnerability, Table 2 shows example disadvantageous pain policies or practices and aligns each with an example corrective action and example monitoring metric.

**TABLE 2:** Example disadvantageous pain policies/practices, corrective actions and monitoring metrics.

Example disadvantageous pain policy/practice	Corrective action	Monitoring metric
Parental exclusion during non‐urgent painful procedures in the Neonatal Intensive Care Unit (NICU)	Implement parent‐inclusive procedural care policy	% of eligible procedures, where at least one parent was present
Default reliance on proxy reports without offering supported self‐report for children with communication disabilities	Introduce validated modified assessment tools or Augmentative and Alternative Communication (AAC)‐based self‐report tools and train clinicians in their use	% of children with complex communication needs offered an AAC‐supported pain assessment
Lack of standardized analgesia protocols for common NICU procedures (e.g., heel lance, venipuncture)	Develop and implement evidence‐based analgesia order sets (sucrose, breastfeeding, skin‐to‐skin)	% of eligible procedures with documented use of at least one recommended analgesic intervention
Restrictive research eligibility criteria excluding children with developmental disabilities	Revise research protocols to include children with disabilities using adapted consent/assent processes and modified and accessible assessment tools	% of active pediatric pain studies that report inclusion of children with developmental disabilities
Inaccessible pain education materials for families (e.g., no easy‐read or multilingual resources)	Co‐design inclusive educational materials with families and distribute in multiple formats	% of families receiving accessible pain education resources upon admission

### 1.9 Addressing Vulnerabilities to Pain

#### 1.9.1 Sick and Preterm Infants

Single, multi‐level, national and international organizational initiatives have been put into place aiming at widespread dissemination of evidence, adoption of evidence‐based recommendations and system‐wide implementation strategies to improve pain management in vulnerable newborns. A list of such initiatives was presented by Harrison and Bueno in their *Pain Clinical Updates* from the International Association for the Study of Pain as part of the *2022 Global Year for Translating Pain Knowledge to Practice* [70]. Since then, the Canadian National Pain standards have been published [71], which speaks to recommendations from the Lancet Child & Adolescent Health Commission [72] transformative goals to make pain: *matter; understood; visible and better*. Such initiatives, coupled with an increasing focus to support parent participation in all aspects of NICU care [55, 73] should bring about the much needed focus on improving pain care in vulnerable sick and preterm newborns.

#### 1.9.2 Children With Developmental Disabilities and Complex Communication Needs

In the past, a lack of appropriate tools to assess pain for the range of communication, cognitive, and physical abilities seen in the cerebral palsy population, and an assumption that many children are unable to self‐report have contributed to inadequate pain assessment. Self‐report is always recommended due to the subjective nature of chronic pain; however, the ability to self‐report may be limited or absent, depending on the severity of an individual child's condition [46]. Where self‐report is deemed not possible, observational tools and proxy reporting tools can be utilized. Although many available tools have proxy as well as self‐report versions, discrepancies between self and proxy‐report have been noted when assessing the impact of pain on emotional functioning and interference [74]. Effective pain management for children with developmental disabilities has been hampered by an inability to easily explore the underlying pain mechanisms due to the compromised ability of individuals to self‐report [46].

Recent advances have improved the way chronic pain is identified and assessed in children and young people with cerebral palsy. A core outcome set has been developed through identifying assessment tools that focus on pain interference and impact on emotional functioning that are valid and feasible for children with cerebral palsy with diverse cognitive and communication abilities [75]. A decision tree to help health professionals implement these tools into clinical care complements this core outcome set [76]. In addition, two of the highest‐rated tools have been adapted for children and young people with cerebral palsy to ensure accessibility and applicability for this population [77].

For children with complex communication needs, we need to think laterally and provide extra time and scaffolding to facilitate self‐report and reduce the burden on the child and family. Many children with complex communication needs use AAC to facilitate their communication. The types of AAC used can range from “low tech” communication boards/books to “high tech” platforms and devices [78]. Allowing sufficient time to ensure those that use AAC can adequately report their own experiences is paramount for equitable care. The current pain‐related vocabulary within these systems has not been informed by people with lived experience and does not adequately address the complexity of pain and its impact on functioning for these children [41]. This is an area for future research. Incorporating methods such as Talking Mats [79], a tool which facilitates expression of opinion, choices, ideas, and preferences using a visual framework, may assist children and young people with a range of developmental disabilities to report the impact pain is having on their everyday lives. Talking Mats Ltd. versions of the newly adapted Pain Interference Questionnaire for Cerberal Palsy and the Fear of Pain Questionnaire adapted for Cerebral Palsy are now freely available to help with this scaffolding [80].

## 2 Conclusion

This article offers a theoretical approach to vulnerability (inherent, situational, pathogenic) that we argue is a usable scaffold for pain clinicians, researchers, and policy makers. Through examples in NICU practice and developmental disabilities, this article translates theoretical ethics into operational concerns (e.g., procedural pain load, parent exclusion, communication inequities), bridging theory to care delivery. We have used Goodin's principle to ground duties of protection and extended it to pain research and routine clinical practice. The content on parent‐delivered analgesia (breastfeeding, skin‐to‐skin, sucrose) and alternative communication gives concrete, evidence‐aligned avenues to mitigate vulnerability. The emphasis on co‐design and accessibility of patient‐and family‐reported outcome measures highlights equity in pain assessment and management. We believe that referencing system‐level initiatives, such as pain standards or family‐centred care, positions this article within current implementation agendas and could aid readers in envisioning pathways to scale.

Human beings are inherently vulnerable to pain. Specific factors can exacerbate the vulnerability to pain in individuals and groups. There is a clinical obligation to reduce vulnerability to pain. Understanding the sources of vulnerability to pain, and how they overlap, can allow more careful discrimination among different sources and kinds of *more than normal* vulnerability to pain. Situational vulnerabilities due to critical illness or complex communication needs need to be considered in order to minimize their impact on the wellbeing of sick and preterm infants and children with developmental disabilities. The possibilities of pathogenic vulnerabilities should be observed in an effort to minimize the harm and exploitation of already vulnerable infants and children. Researchers, health professionals, and policy makers must consider all sources of vulnerability to pain when designing and implementing solutions to minimize pain and its adverse impacts.

## Funding

The authors have nothing to report.

## Conflicts of Interest

The authors declare no conflicts of interest.

## Data Availability

The authors have nothing to report.
